# A novel reshapable catheter facilitates selective biliary and pancreatic duct cannulation following endoscopic submucosal dissection including papilla

**DOI:** 10.1055/a-2598-5509

**Published:** 2025-05-26

**Authors:** Haruo Miwa, Kingo Hirasawa, Kazuki Endo, Ritsuko Oishi, Yuichi Suzuki, Hiromi Tsuchiya, Shin Maeda

**Affiliations:** 1 26437 Gastroenterological Center, Yokohama City University Medical Center, Yokohama, Japan; 2 26437 Division of Endoscopy, Yokohama City University Medical Center, Yokohama, Japan; 3 Department of Gastroenterology, Yokohama City University Graduate School of Medicine, Yokohama, Japan

Endoscopic submucosal dissection including the papilla (ESDIP) carries the risk of delayed bleeding and perforation 1 2 ; therefore, endoscopic nasobiliary and nasopancreatic duct drainage (ENBPD) is recommended to prevent adverse events 3 4 . However, after ESDIP, the maneuverability of the duodenoscope is restricted, making it difficult to align with the axes of the bile duct and pancreatic ducts for cannulation. A novel reshapable catheter (VEGA; Japan Lifeline Co., Ltd., Tokyo, Japan) features a coiling shaft that allows for easy manual adjustment to an appropriate angle ( Fig. 1 ).

**Fig. 1:**
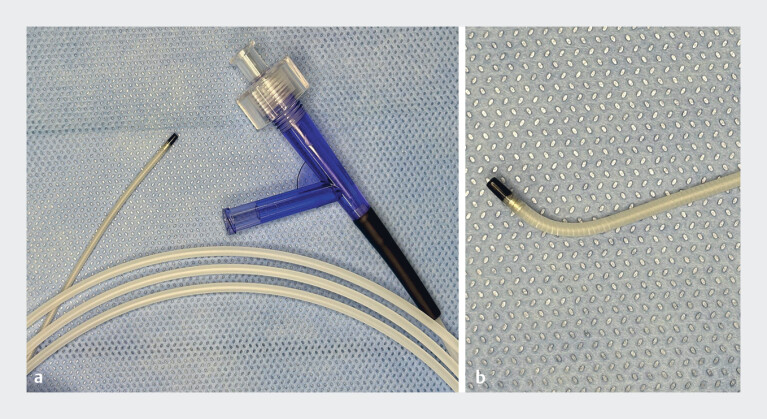
The novel reshapable catheter (VEGA; Japan Lifeline Co., Ltd., Tokyo Japan). **a** The catheter features a coiling shaft. **b** The tip of the catheter allows for easy manual adjustment to an appropriate angle.

A 41-year-old woman was referred to our hospital because of a laterally spreading tumor involving the papilla. ENBPD was planned to prevent adverse events following ESDIP ( Fig. 2 , Video 1 ). A duodenoscope (TJF-290V; Olympus Medical Systems, Tokyo, Japan) was inserted into the second part of the duodenum ( Fig. 3 ). The duodenal lumen was narrowed due to clipping performed during ESDIP. The orifices of the bile and pancreatic ducts were identified beyond the clips. The axis of the pancreatic duct ran downward. First, the novel reshapable catheter was used in a straight shape. Pancreatography was successfully performed before guidewire insertion. The bile duct axis appeared to run upward. The catheter was manually reshaped to adjust to the direction of the bile duct. The tip of the catheter was gently positioned at the orifice of the bile duct and the guidewire was successfully inserted. Cholangiography confirmed successful bile duct cannulation ( Fig. 4 ). Subsequently, nasopancreatic duct and nasobiliary tubes were placed, and the duodenoscope was carefully withdrawn. The patient experienced no complications, and she was discharged on Day 8.

**Fig. 2:**
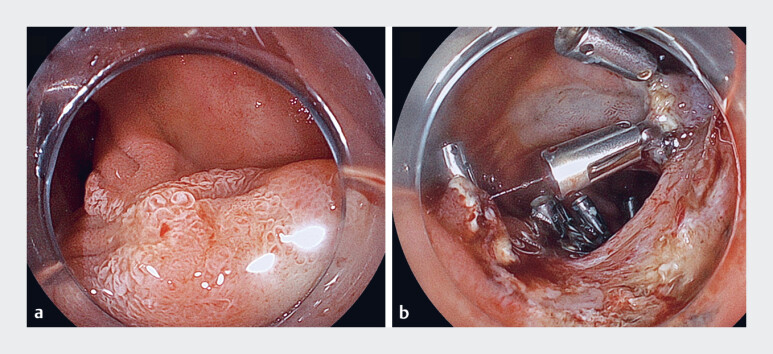
Endoscopic images. **a** A laterally spreading tumor involving the papilla. **b** After endoscopic submucosal dissection, the duodenal lumen was narrowed due to clipping.

**Fig. 3:**
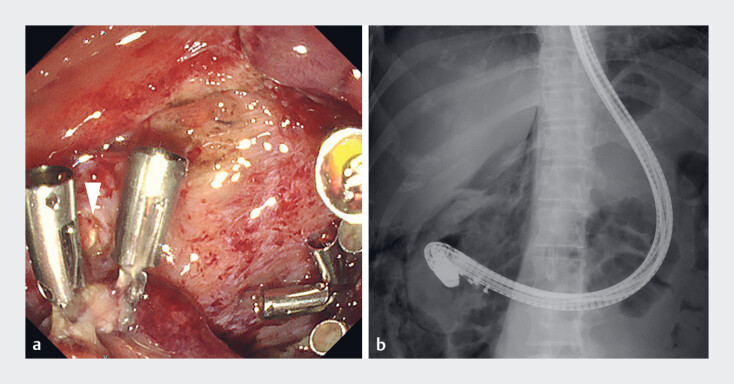
After endoscopic submucosal dissection including the papilla. **a** The orifices of the bile and pancreatic ducts were identified beyond the clips (arrowhead). **b** The maneuverability of the duodenoscope was restricted.

**Fig. 4:**
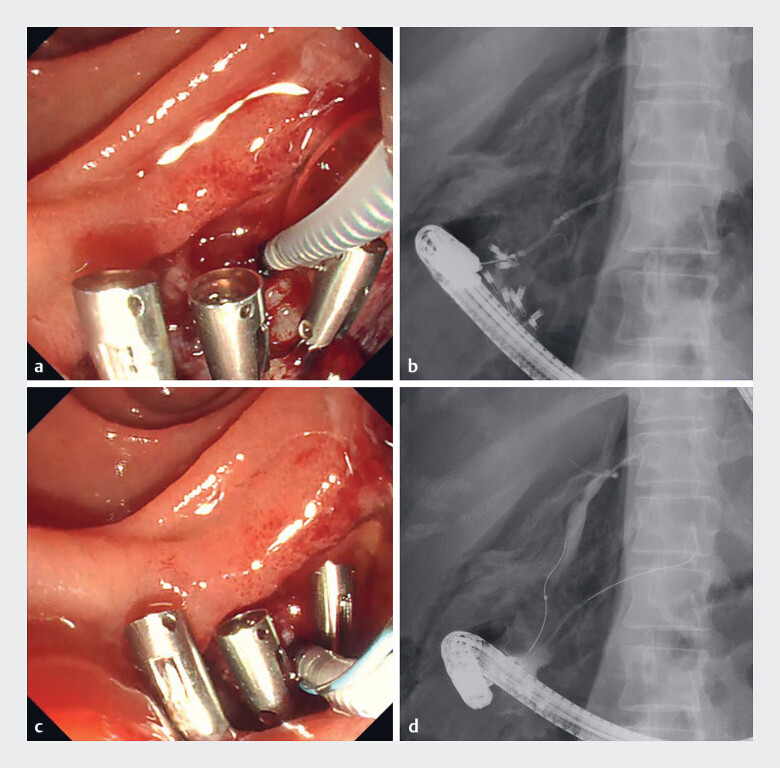
Selective biliary and pancreatic duct cannulation. **a** The axis of the pancreatic duct ran downward. The catheter was used in a straight shape. **b** Pancreatic duct cannulation was successfully performed. **c** The bile duct axis appeared to run upward. The catheter was manually reshaped to adjust to the direction of the bile duct. **d** Cholangiography confirmed successful bile duct cannulation.

The novel reshapable catheter was useful for biliary and pancreatic duct cannulation after endoscopic submucosal dissection including the papilla.Video 1

To the best of our knowledge, this is the first report describing the use of a novel reshapable catheter for selective biliary and pancreatic duct cannulations. This catheter facilitates cannulation in challenging cases where duodenoscope maneuverability is restricted.

Endoscopy_UCTN_Code_TTT_1AR_2AC

## Notes

Endoscopy E-Videos https://eref.thieme.de/e-videos
*E-Videos* is an open access online section of the journal *Endoscopy* , reporting on interesting cases and new techniques in gastroenterological endoscopy. All papers include a high-quality video and are published with a Creative Commons CC-BY license. Endoscopy E-Videos qualify for HINARI discounts and waivers and eligibility is automatically checked during the submission process. We grant 100% waivers to articles whose corresponding authors are based in Group A countries and 50% waivers to those who are based in Group B countries as classified by Research4Life (see: https://www.research4life.org/access/eligibility/ ). This section has its own submission website at https://mc.manuscriptcentral.com/e-videos .
